
JOURNAL INFORMATION
==============================
NLM Title Abbreviation: Open Forum Infect Dis
Journal ID: Open Forum Infect Dis
NLM Journal ID: 101637045
Journal ID: ofid

Open Forum Infectious Diseases

EISSN: 2328-8957
Publisher: Oxford University Press

ARTICLE INFORMATION
==============================
PMCID: PMC10485881
PMID: 37691735
DOI: 10.1093/ofid/ofad456
Article ID: ofad456
Article version: 1
Subjects: Insight Article, AcademicSubjects/MED00290

Two-year durability of REBYOTA™ (RBL), a live biotherapeutic for the prevention of recurrent Clostridioides difficile infections

Orenstein Robert Division of Infectious Diseases, Mayo Clinic, Phoenix, AZ, USA

Hecht Gail Division of Gastroenterology, Hepatology and Nutrition, Loyola University Medical Center, Maywood, IL, USA

Harvey Adam Rebiotix Inc., a Ferring Company, Roseville, MN, USA

Tillotson Glenn GST Micro LLC, North, VA

Khanna Sahil Division of Gastroenterology and Hepatology, Mayo Clinic, Rochester, MN, USA

corresponding author - khanna.sahil@mayo.edu
Publication date: 2023 Sep
Electronic publication date: 2023 Sep 8
Volume: 10
Issue: 9
Electronic Location ID: ofad456
2023 Sep 8
Copyright: © The Author(s) 2023. Published by Oxford University Press for the Infectious Diseases Society of America.
Copyright year: 2023
License: This is an Open Access article distributed under the terms of the Creative Commons Attribution License (https://creativecommons.org/licenses/by/4.0/), which permits unrestricted reuse, distribution, and reproduction in any medium, provided the original work is properly cited.
License URL: https://creativecommons.org/licenses/by/4.0/

Keywords: Clostridioides difficile infection, live biotherapeutic product, durable response, microbiome, bile acids
Page count: 6

==============================

Open Forum Infectious Diseases

Open Forum Infectious Diseases 2023; 10: 9
doi: 10.1093/ofid/ofad456

InSight

### Two-year durability of REBYOTA™ (RBL), a live biotherapeutic for the prevention of recurrent *Clostridioides difficile* infections

Robert Orenstein [1], Gail Hecht [2], Adam Harvey [3], Glenn Tillotson [4], Sahil Khanna* [5]

[1] Division of Infectious Diseases, Mayo Clinic, Phoenix, AZ, USA
[2] Division of Gastroenterology, Hepatology and Nutrition, Loyola University Medical Center, Maywood, IL, USA
[3] Rebiotix Inc., a Ferring Company, Roseville, MN, USA
[4] GST Micro LLC, North, VA
[5] Division of Gastroenterology and Hepatology, Mayo Clinic, Rochester, MN, USA (*corresponding author – khanna.sahil@mayo.edu)

**Keywords:** *Clostridioides difficile* infection, live biotherapeutic product, durable response, microbiome, bile acids

### Introduction

*Clostridioides difficile infection* (CDI), the leading cause of antibiotic- and healthcare-associated infectious diarrhea in the United States, is associated with almost half a million infections annually [1, 2]. Dysbiosis, a perturbation in the intestinal microbiota composition characterized by reduced richness and diversity in the gut microbiome is implicated in CDI [3-5]. While antibiotics are the standard treatment for CDI [6], they often lead to microbial dysbiosis that predisposes patients to recurrence [7]; between 20–35% of patients experience recurrent CDI (rCDI) [8], of which up to 60% experience subsequent recurrences [9]. Recurrence leads to significant morbidity and mortality along with substantial economic and quality of life implications that increase with each subsequent recurrence [10].

Most CDI recurrences occur rapidly within a few weeks of antibiotic completion [11, 12]. However, there is a paucity of data regarding the incidence of CDI events over more than a few months. Data from the Premier Healthcare Database showed that some patients experienced up to five or more episodes in one year [13]. Given that antibiotics are not always effective at achieving a sustained cure, additional treatments that repair the microbiome after discontinuation of antibiotic treatment are needed for patients with rCDI.

A common clinical scenario is patients who develop rCDI due to repeated antibiotic exposure and are at risk of getting ongoing antibiotics leading to further recurrences. We discuss the representative case of a 78-year-old female with a history of depression, hypercholesterolemia, obesity, *C. difficile* infection a few years ago, and recurrent bacterial sinusitis for which she recently received 875/125 mg amoxicillin/clavulanate twice daily (BID) for 10 days. Her sinus symptoms improved but three days later she developed diarrhea. A stool sample for *C. difficile* toxin testing was positive and she was initially prescribed fidaxomicin 200 mg BID for 10 days but could not fill the prescription due to cost, so she was given vancomycin 125 mg four times daily (QID) for 10 days. Symptoms improved but 10 days later the diarrhea returned with moderate abdominal pain. The patient was briefly admitted to the hospital and given a vancomycin taper: 125 mg QID, followed by 125 mg BID for seven days, then one capsule daily for seven days, then one capsule every other day for 14 days. Her stools returned to normal both in frequency and consistency. One month later her diarrhea returned which was again *C. difficile* toxin positive. She refused fidaxomicin again due to cost and was prescribed another vancomycin taper and pulse regimen. She was distressed with her experience and was wondering about recurrence prevention strategies due to multiple recurrences of CDI in a short period.

As a clinician you discuss microbiota-based therapies including fecal microbiota transplantation and an available live biotherapeutic product (LBP), Fecal microbiota Live – jslm (RBL / Rebyota™). She is interested in long term efficacy and safety of these therapeutics.

### Fecal Microbiota Transplantation (FMT)

FMT has emerged as a well-recognized strategy for the management of rCDI by restoring gut microbiota homeostasis [14]. CDI treatment guidelines published by both the Infectious Diseases Society of America and the American College of Gastroenterology now endorse FMT as a recommended therapy for rCDI unresponsive after two or more recurrences treated with antibiotics [6, 15].

One multicenter retrospective study investigated the efficacy and safety of a FMT protocol that utilized a mix of fresh or frozen stool

© The Author(s) 2023. Published by Oxford University Press for the Infectious Diseases Society of America. This is an Open Access article distributed under the terms of the Creative Commons Attribution License (https://creativecommons.org/licenses/by/4.0/), which permits unrestricted reuse, distribution, and reproduction in any medium, provided the original work is properly cited.

obtained from patient-directed and universal donors with recognized donor selection and screening processes. The FMT protocol achieved an 89.4% short-term cure rate (absence of CDI at ≤8 weeks), an 85.5% 6-month cure rate (absence of CDI at ≤6 months), and a 75.8% sustained cure (the absence of CDI at any timepoint after FMT). Half of these patients reported new medical conditions or diagnoses post-FMT, but the majority were considered unlikely to be related to the treatment. Furthermore, no new serious infections developed and there was no clustering of diseases associated with dysbiosis such as obesity, hyperlipidemia, and hypertension [16].

There are several challenges with FMT, these include lack of standardization of the product processing, burden of comprehensive pathogen screening and donor availability. These issues led to the development of standardized biologic therapeutic products which avoided these issues. The first live biotherapeutic product, REBYOTA™ , was approved by the US Food and Drug administration (FDA) in November 2022.

### Live biotherapeutic products (LBP)

While the Centers for Disease Control and Prevention and the United States FDA consider CDI treatment success as prevention of recurrence for eight weeks after treatment [17], data on the long-term success of CDI treatments is lacking. Determining whether these products can maintain efficacy beyond six months is important to determine whether they can provide a long-term solution to the problem of recurrence of CDI.

### REBYOTA™ (RBL)

RBL is an FDA-approved, rectally administered, human-derived, broad consortium microbiota-based LBP for rCDI. A phase 3 trial of a single dose of RBL demonstrated efficacy of 70.6% at week 8 in subjects with ≥2 episodes of CDI and >90% of subjects with treatment success had sustained response through six months [18].

Long-term efficacy and safety data are available up to two years for RBL in adults (≥18 years old) with a diagnosis of rCDI and either ≥ 2 recurrences of CDI following ≥2 rounds standard-of-care antibiotic therapy or ≥2 episodes of severe CDI requiring hospitalization [19, 20].

In the PUNCH CD Open Label Phase 2 trial, subjects who received two doses of RBL demonstrated a 78.9% treatment success rate at 56 days post-treatment compared to 30.7% for the historical control group ($p < 0.0001$). Among the RBL responders, 97% remained CDI-free at six months while 95% and 91% remained occurrence-free at one and two years, respectively [19]. Post-hoc sub-analyses demonstrated sustained treatment response through six months, one year, and two years after treatment for 98%, 98%, and 94% of patients <65 years of age, respectively, and 97%, 93%, and 88% of patients ≥65 years of age. Similarly, 98%, 96%, and96% of patients with 2 or 3 prior CDI episodes, respectively, and 97%, 94%, and 86% of patients with ≥4 episodes remained recurrence-free at six months, one year, and two years. Similar sustained response rates were also demonstrated in patients categorized by gender and race [21].

Throughout the study period, most TEAEs were mild to moderate (84%), primarily related to pre-existing conditions (58%), and were experienced during the first four weeks after treatment (60%). The most common adverse events were gastrointestinal in nature with diarrhea reported in 30% of subjects. Importantly, there were no reported infections caused by pathogens traceable to RBL. While 13% of subjects experienced urinary tract infections, none were deemed related to RBL or the enema procedure. Only two subjects reported serious adverse events assessed as possibly related to RBL, including ileus, leukocytosis, pyrexia, atrial fibrillation, coincident with severe CDI eventually leading to death in one subject and three CDI episodes (one severe) in the other subject; but these were also deemed related to pre-existing conditions and CDI [19].

In the PUNCH CD2 randomized, placebo-controlled Phase 2b trial, subjects were randomized 1:1:1 to receive two doses of RBL, two doses of placebo, or one dose of RBL followed by one dose of placebo. The primary efficacy endpoint was treatment success after two doses of RBX2660 compared with two doses of placebo. While the prespecified intent-to-treat efficacy analysis was not statistically significant [22], in the final per-protocol population analysis, treatment success was achieved in 87.5% of subjects who received one dose of RBL followed by one dose of placebo versus 58.1% in the placebo-only group ($p = 0.017$). There was no significant difference in treatment response between the two doses of RBL (75%) and placebo ($p = 0.17$) [20]. Among the RBL responders in the two-dose group, 100% remained CDI-free at three months whereas 91.7% of the two dose RBL group remained recurrence-free at six months and 87.5% remained recurrence-free at both one and two years. The one dose RBL group achieved a more sustained clinical response as 95.7% remained recurrence-free at at six months, one year, and two years (data on file, Ferring Pharmaceuticals).

The safety profile of RBL, whether delivered as one or two doses, was like the placebo group; most (87.6%) of the TEAEs were mild or moderate in severity, and 32.5% were primarily related to gastrointestinal disorders. Three (2.3%) serious adverse events (constipation, recurrent acute myeloid leukemia, abdominal pain) were reported as possibly related to RBL. There were no reports of bacteremia or fungemia over the two-year period [20].

RBL has been shown to effectively restore dysbiosis within the gut microbiome. After administration, the gut microbiome in RBL responders becomes more similar to the unperturbed microbiome of RBL, and hence more closely resembles the microbiome of healthy patients. Microbiota changes occur within seven days of treatment and last for six months to two years; treatment not only increases alpha diversity, but it also significantly increases *Bacteroidia* and *Clostridia*, which are restored to predominance, and decreases *Gammaproteobacteria*, *Bacilli*, and *Erysipelotrichia* [19, 23-25].

Restoration of microbial diversity and higher secondary:primary bile acid ratios are associated with a continued CDI-free state in RBL-treated subjects
BASELINE (post-CDI)
Relative abundance
0.0
0.2
0.4
0.6
0.8
1.0
Microbiota
Bacteriodia
Clostridia
Gammaproteobacteria
0%
25%
50%
75%
100%
Bile acids (relative percentage)
The microbiome is dysbiotic based on relative abundance of Gammaproteobacteria vs Firmicutes and Bacteroidetes
Treatment with RBL
8W
79 to 88%*
*clinical efficacy at time point
Relative abundance
Microbiota
Bacteriodia
Clostridia
Gammaproteobacteria
Bile acids (relative percentage)
6M
92 to 97%*
Relative abundance
Microbiota
Bacteriodia
Clostridia
Gammaproteobacteria
Bile acids (relative percentage)

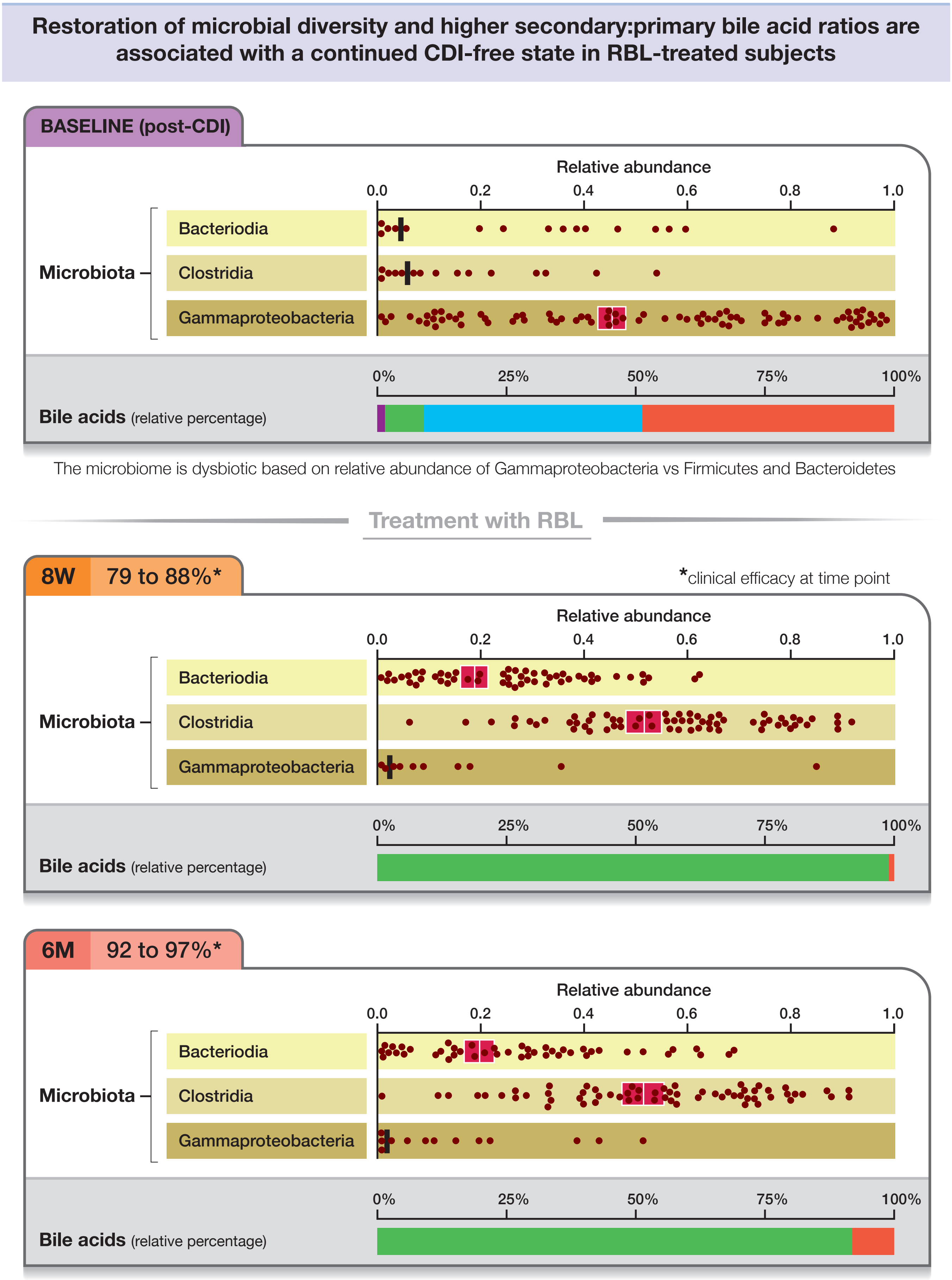

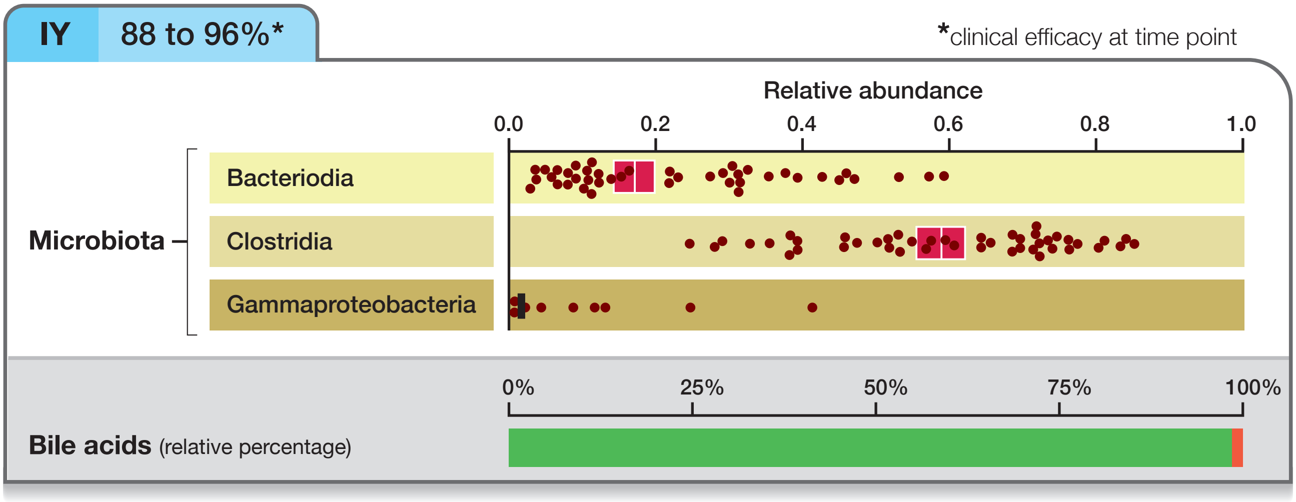

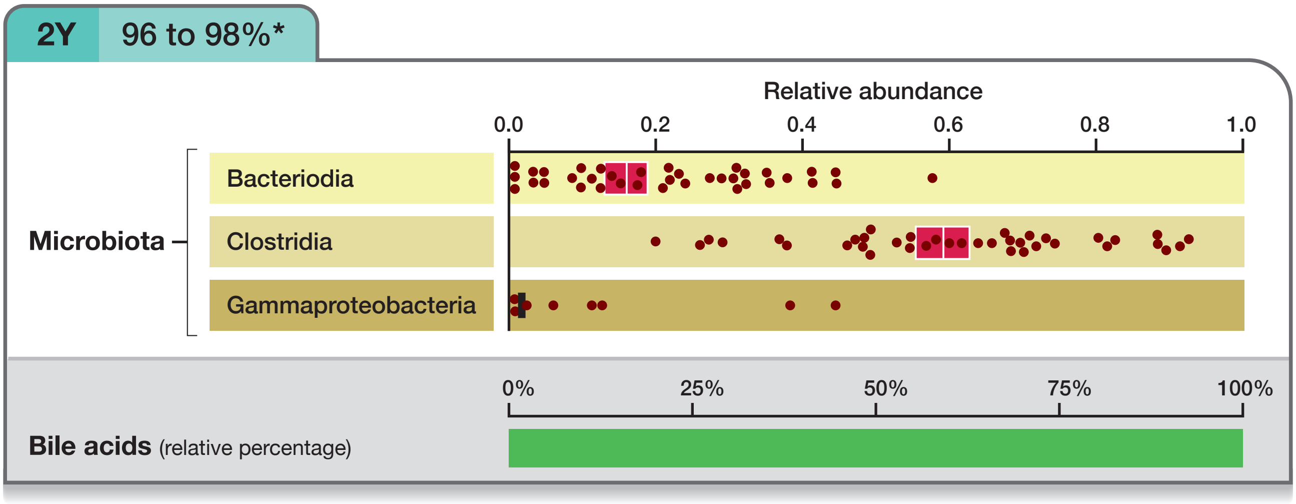

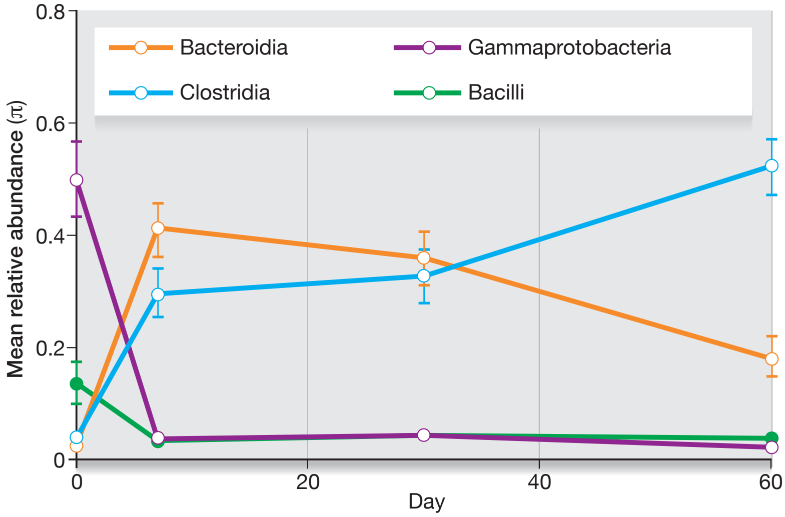

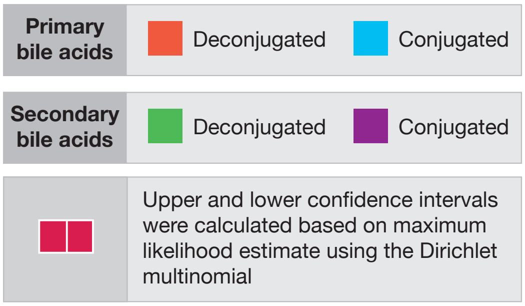

**1.** Orenstein R, Dubberke ER, Khanna S, et al. *Durable reduction of Clostridioides difficile infection recurrence and microbiome restoration after treatment with RBX2660: results from an open-label phase 2 clinical trial.* BMC Infect Dis. 2022;22(1):245; **2.** Dubberke, E.R., et al., *Final Results from a Phase 2b Randomized, Placebo-Controlled Clinical Trial of RBX2660: A Microbiota-Based Drug for the Prevention of Recurrent Clostridioides difficile Infection.* Infect Dis Ther, 2023. 12(2): p. 703-709; **3.** Data on file, Ferring Pharmaceuticals; **4.** Papazyan R, Ferdyan N, Srinivasan K, et al. *Human Fecal Bile Acid Analysis after Investigational Microbiota-Based Live Biotherapeutic Delivery for Recurrent Clostridioides difficile Infection.* Microorganisms. 2023;11(1):135.

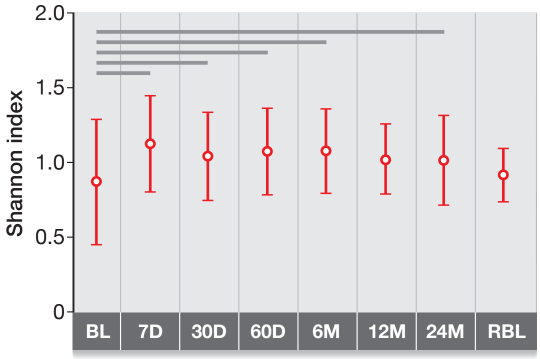

RBL restores bile acid metabolism towards healthier bile acid profiles characterized by higher secondary:primary bile acid ratios. A bile acid composition enriched with secondary bile acids is normally seen in the healthy gut whereas enrichment with primary bile acids, as seen in baseline in Figure 1, is associated with dysbiosis and an increased risk of CDI recurrence [26, 27]. To quantify mean changes to bile compositions induced by RBL treatment, bile acids were categorized into primary conjugated, primary deconjugated, secondary deconjugated, and secondary conjugated. After RBL administration, Figure 1 illustrates a significant drop in primary bile acids concurrent with increased secondary bile acids that increased over time plateauing after eight weeks and remaining sustained throughout two years after treatment [26]. The shifts in secondary versus primary bile acids support the continued efficacy over two years.

### Case resolution

Upon discussion of options, she opted for fecal microbiota Live – jslm (RBL /REBYOTA™) due to the non-invasive nature of the product and the long-term safety and efficacy data available. She underwent rectal administration after a wash out period of 48 hours after vancomycin and did not experience any difficulty with the administration and had no adverse reactions.

### Conclusions

Microbiota-directed approaches for preventing CDI recurrences can provide both short- and long-term benefit. The data thus far suggests that the first FDA-approved LBP RBL can prevent rCDI in initial responders for up to two years after treatment administration.

Clinical reduction of CDI recurrence is correlated with restorative microbiome changes, including significantly increased alpha diversity as well as an increased abundance of *Bacteroidia* and *Firmicutes* including Clostridia-class bacteria and a decreased abundance of *Gammaproteobacteria* and *Bacilli*.

Healthier bile acid profiles, including significantly reduced primary bile acids and restored secondary bile acids are also correlated with clinical reduction of CDI recurrence.

Corresponding author name: **Dr. Sahil Khanna** Division of Gastroenterology and Hepatology, Mayo Clinic, Rochester, MN, USA – khanna.sahil@mayo.ed

InSight title: **Two-year durability of Rebyota (RBL), a live biotherapeutic for the prevention of recurrent *Clostridioides difficile* infections**

Sponsor: This InSight was sponsored by **Ferring Pharmaceuticals.**

DOI: https://10.1093/ofid/ofad456

OXFORD UNIVERSITY PRESS

Open Forum Infectious Diseases

© The Author(s) 2023. Published by Oxford University Press for the Infectious Diseases Society of America. This is an Open Access article distributed under the terms of the Creative Commons Attribution License (https://creativecommons.org/licenses/by/4.0/), which permits unrestricted reuse, distribution, and reproduction in any medium, provided the original work is properly cited.

### References

1. Lessa, F.C., et al., *Burden of Clostridium difficile infection in the United States.* N Engl J Med, 2015. **372**(9): p. 825-34.
2. Magill, S.S., et al., *Changes in Prevalence of Health Care-Associated Infections in U.S. Hospitals.* N Engl J Med, 2018. **379**(18): p. 1732-1744.
3. Shahinas, D., et al., *Toward an understanding of changes in diversity associated with fecal microbiome transplantation based on 16S rRNA gene deep sequencing.* mBio, 2012. **3**(5).
4. Chang, J.Y., et al., *Decreased diversity of the fecal Microbiome in recurrent Clostridium difficile-associated diarrhea.* J Infect Dis, 2008. **197**(3): p. 435-8.
5. Seekatz, A.M., et al., *Dynamics of the fecal microbiome in patients with recurrent and nonrecurrent Clostridium difficile infection.* Genome Med, 2016. **8**(1): p. 47.
6. McDonald, L.C., et al., *Clinical Practice Guidelines for Clostridium difficile Infection in Adults and Children*: 2017 Update by the Infectious Diseases Society of America (IDSA) and Society for Healthcare Epidemiology of America (SHEA). Clin Infect Dis, 2018. **66**(7): p. e1-e48.
7. Seekatz, A.M., N. Safdar, and S. Khanna, *The role of the gut microbiome in colonization resistance and recurrent Clostridioides difficile infection.* Therap Adv Gastroenterol, 2022. **15**: p. 17562848221134396.
8. Cornely, O.A., et al., T*reatment of first recurrence of Clostridium difficile infection: fidaxomicin versus vancomycin.* Clin Infect Dis, 2012. **55 Suppl 2**: p. S154-61.
9. Nelson, W.W., et al., *Health care resource utilization and costs of recurrent Clostridioides difficile infection in the elderly: a real-world claims analysis.* J Manag Care Spec Pharm, 2021. **27**(7): p. 828-838.
10. Feuerstadt, P., N. Theriault, and G. Tillotson, *The burden of CDI in the United States: a multifactorial challenge.* BMC Infect Dis, 2023. **23**(1): p. 132.
11. Vardakas, K.Z., et al., *Treatment failure and recurrence of Clostridium difficile infection following treatment with vancomycin or metronidazole: a systematic review of the evidence.* Int J Antimicrob Agents, 2012. **40**(1): p. 1-8.
12. Abujamel, T., et al., *Defining the vulnerable period for re-establishment of Clostridium difficile colonization after treatment of C. difficile infection with oral vancomycin or metronidazole.* PLoS One, 2013. **8**(10): p. e76269.

13. Tillotson, G., S. Ng, and A. Guo, *Clostridioides difficile: a recurrent infection with broad economic consequences for US hospitals [abstract 4-035 ], in In: ASHP Conference for Pharmacy Leaders;* December 2022, Las Vegas NV .

14. Shao, T., et al., *The Evolving Landscape of Fecal Microbial Transplantation*. Clin Rev Allergy Immunol, 2023: p. 1-20.

15. Kelly, C.R., et al., *ACG Clinical Guidelines: Prevention, Diagnosis, and Treatment of Clostridioides difficile Infections*. Am J Gastroenterol, 2021. **116**(6): p. 1124-1147.

16. Perler, B.K., et al., *Long-Term Efficacy and Safety of Fecal Microbiota Transplantation for Treatment of Recurrent Clostridioides difficile Infection*. J Clin Gastroenterol, 2020. 54(8): p. 701-706.

17. CDC. *Clostridioides difficile Infection (CDI) Tracking*. 2022 [cited 2023 05 March]; Available from: https://www.cdc.gov/hai/eip/cdiff-tracking.html.

18. Khanna, S., et al., *Efficacy and Safety of RBX2660 in PUNCH CD3, a Phase III, Randomized, Double-Blind, Placebo-Controlled Trial with a Bayesian Primary Analysis for the Prevention of Recurrent Clostridioides difficile Infection*. Drugs, 2022.

19. Orenstein, R., et al., *Durable reduction of Clostridioides difficile infection recurrence and microbiome restoration after treatment with RBX2660: results from an open-label phase 2 clinical trial*. BMC Infect Dis, 2022. **22**(1): p. 245.

20. Dubberke, E.R., et al., *Final Results from a Phase 2b Randomized, Placebo-Controlled Clinical Trial of RBX2660: A Microbiota-Based Drug for the Prevention of Recurrent Clostridioides difficile Infection*. Infect Dis Ther, 2023. **12**(2): p. 703-709.

21. Orenstein, R., et al., *24-Month Sustained Clinical Response and Safety of RBX2660 in Participants with Recurrent Clostridioides difficile Infection: Subgroup Analysis [poster], in In: ACG; October 21-26. Charlotte, North Carolina.* 2022.

22. Dubberke, E.R., et al., *Results From a Randomized, Placebo-Controlled Clinical Trial of a RBX2660-A Microbiota-Based Drug for the Prevention of Recurrent Clostridium difficile Infection.* Clin Infect Dis, 2018. **67**(8): p. 1198-1204.

23. Blount, K., et al., *Treatment success in reducing recurrent Clostridioides difficile infection with investigational live biotherapeutic RBX2660 is associated with microbiota restoration: Consistent evidence from a Phase 3 clinical trial.* Open Forum Infect Dis, 2021. **8**: p. S624-S625.

24. Blount, K.F., et al., *Restoration of Bacterial Microbiome Composition and Diversity Among Treatment Responders in a Phase 2 Trial of RBX2660: An Investigational Microbiome Restoration Therapeutic.* Open Forum Infect Dis, 2019. **6**(4): p. ofz095.

25. Kwak, S., et al., *Impact of investigational microbiota therapeutic RBX2660 on the gut microbiome and resistome revealed by a placebo-controlled clinical trial.* Microbiome, 2020. **8**(1): p. 125.

26. Papazyan, R., et al., *Human Fecal Bile Acid Analysis after Investigational Microbiota-Based Live Biotherapeutic Delivery for Recurrent Clostridioides difficile Infection.* Microorganisms, 2023. **11**(1).

27. Papazyan, R., et al., *Rapid restoration of bile acid compositions after treatment with RBX2660 for recurrent Clostridioides difficile infection—Results from the PUNCH CD3 Phase 3 Trial.* Open Forum Infect Dis, 2021. **8**(Suppl 1): p. S610.

**Conflict of Interest Statement:**

**Robert Orenstein** is an employee of the Division of Infectious Diseases, Mayo Clinic in Arizona, 5777 e Mayo Blvd, Phoenix, AZ 85054, USA, **Gail Hecht** is an employee of the Division of Gastroenterology, Hepatology and Nutrition, Loyola University Medical Center, Maywood, IL, USA., **Adam Harvey** is an employee of Rebiotix Inc., a Ferring Company, Roseville, MN, USA, **Glenn Tillotson** is a consultant to Ferring Pharmaceuticals and Spero Therapeutics, **Sahil Khanna** is an employee the Division of Gastroenterology and Hepatology, Mayo Clinic, Rochester, MN, USA.
